# Contagious risk taking: social information and context influence wild jackdaws’ responses to novelty and risk

**DOI:** 10.1038/srep27764

**Published:** 2016-06-10

**Authors:** Alison L. Greggor, Guillam E. McIvor, Nicola S. Clayton, Alex Thornton

**Affiliations:** 1 Department of Psychology, University of Cambridge, Cambridge, CB2 3EB, UK; 2 Centre for Ecology and Conservation, University of Exeter, Penryn Campus, TR10 9FE, UK

## Abstract

Although wild animals increasingly encounter human-produced food and objects, it is unknown how they learn to discriminate beneficial from dangerous novelty. Since social learning allows animals to capitalize on the risk-taking of others, and avoid endangering themselves, social learning should be used around novel and unpredictable stimuli. However, it is unclear whether animals use social cues equally around all types of novelty and at all times of year. We assessed whether wild, individually marked jackdaws—a highly neophobic, yet adaptable species—are equally influenced by social cues to consume novel, palatable foods and to approach a startling object. We conducted these tests across two seasons, and found that in both seasons observers were more likely to consume novel foods after seeing a demonstrator do so. In contrast, observers only followed the demonstrator in foraging next to the object during breeding season. Throughout the year more birds were wary of consuming novel foods than wary of approaching the object, potentially leading to jackdaws’ greater reliance on social information about food. Jackdaws’ dynamic social cue usage demonstrates the importance of context in predicting how social information is used around novelty, and potentially indicates the conditions that facilitate animals’ adjustment to anthropogenic disturbance.

As humans drastically alter habitats worldwide we create novel stimuli, such as foods and objects. How animals respond to human-created novelty, and whether they learn to distinguish dangerous from beneficial stimuli, may crucially influence their survival and reproductive success^1^,^2^. Yet these stimuli often overlap in ways that make assessing their risk difficult. For example, while animals might be expected to approach certain combinations of novel food and man-made objects (e.g. crisps in a shiny packet on the pavement), we equally expect them to avoid other combinations of food and objects (e.g. reflective crop deterrents in a field). Although making incorrect assessments of these stimuli combinations can have serious consequences (e.g. promoting ecological traps^3^), little is known about how animals learn about the novelty we create.

Social cue usage is favoured when animals are uncertain, or when asocial learning is costly^4^; both of which are characteristic of encountering anthropogenic novelty. Even though theoretical models propose that using social cues helps animals adjust to changing habitats^5^,^6^, it is unclear how well these predictions apply to wild systems. Firstly, the motivation to approach novelty can vary with state^7^, age^8^ or season^9^. For example, birds go through dramatic physiological, and hormonal^10^ changes in preparation for breeding, and often show changes in territoriality, social system, activity levels and caloric demands^11^. Although these factors are likely to have substantial impacts on risk taking, and therefore social cue usage, studies rarely address seasonal differences in ecology and behaviour^12^, nor the cognitive implications such differences may have^13^. Moreover, while seasonal variation in exploration and object neophobia has been documented, the effect and direction of seasonal change in birds is inconsistent (e.g.^9^,^14^,^15^,^16^), and it is unknown how this variation may impact social cue usage.

Secondly, it is unclear whether social information would be favoured to the same extent around different types of risk. Theoretical studies suggest that animals should rely on social cues before consuming novel foods^17^, and empirical ones indicate that social cues are used when deciding whether to approach potentially threatening objects^18^. However, avoidance of these different stimuli types does not always correlate within individuals^19^. Additionally, the same strategies may not be utilized in approaching novel versus threatening stimuli because different cognitive processes underlie novelty perception and risk assessment^20^. For example, the hesitancy that animals may exhibit in approaching novel foods does not always correlate with measures of predatory wariness (e.g.^21^), thereby implying that different levels of risk may be involved in approaching novel versus known threats, or that responses stem from divergent processes such as taste sensitivity versus fear. Therefore, to understand how animals learn to distinguish beneficial from dangerous stimuli, it is critical we examine situations in which wild animals approach and learn about novelty that co-occurs with other forms of risk.

We investigated the influence of social cues on wild birds’ foraging choices in populations where individuals regularly encounter beneficial and dangerous man-made stimuli. Like other birds of the corvid family, jackdaws (*Corvus monedula*) are highly neophobic^22^ and highly innovative^23^,^24^; a seemingly paradoxical combination since neophobia is thought to inhibit innovation^25^,^26^. Jackdaws commonly utilize human resources (e.g. foods and nesting sites), but are legally classified as vermin (Wildlife & Countryside Act 1981) and are targeted by deterrents and active persecution^27^. Consequently, jackdaws provide an excellent system to assess how information about novelty is acquired around risk because their responses towards dangerous and beneficial novelty can determine their survival around humans. This is especially true in the village and farmland sites where we conducted this study, because corvids are culled in the surrounding area as a result of perceived conflict with human interests.

We measured the responses of individually marked, free-flying jackdaws towards novel coloured versus familiar food, in locations closer or farther from a startling camera that flashed when it detected motion. We ensured that food was perceived as novel, and reactions were not due to aversions toward a particular colour, by training different wild jackdaw groups in a series of experimental stages (see Fig. 1). We measured the impact of social information on risk taking by comparing birds’ choices between foraging bouts where other conspecifics (termed “demonstrators”) made risky choices to bouts where demonstrators chose the relatively safer food or location option.

As novel foods and a startling object both pose potential risks, we predicted that jackdaws would rely on social information in guiding their food and foraging location choices. Since jackdaws’ forage in large flocks during winter and forage alongside their mate and members of their breeding colony during the breeding season^28^, they have access to social foraging cues all year. However, we anticipated the effects of social information would change across the year as seasonal influences on motivation and risk aversion would influence social information use. Given that corvids often have increased caloric requirements during the breeding season^29^, and hunger can stimulate risk taking^30^, one may expect that breeding jackdaws would be more willing to take risks, and therefore less reliant on social information. Since social cues would theoretically be more useful in contexts of higher risk, we expected that fewer birds would take risks in approaching either stimulus type at times of year where social information use was highest.

## Results

### Influence of social information

We found that jackdaws were more likely to eat a novel food if a demonstrator had just done so, and this effect was strongest during the *training* stage of the experiment (GLMM, n = 212 visits by 44 individuals, interaction term, Est = 3.13 ± 1.27, z = 2.46, p = 0.014; Fig. 2). Additionally, observers were more likely to eat the novel food when more trials had been run at each table (Est = 0.23 ± 0.11, z = 2.07, p = 0.038), and observers that landed on the risky side of the table showed a marginally significant tendency to avoid eating novel food (Est = −1.21 ± 0.59, z = −2.06, p = 0.040). Season did not have an impact on social cue usage about food, nor did sex, age, or the demonstrator’s proximity to the camera (see Supplementary Table S1).

In contrast, the presence of a demonstrator near the camera only encouraged observers to land on the risky side of the table during breeding season (GLMM, n = 516 visits by 85 birds, interaction term, Est = 0.86 ± 0.42, z = 2.06, p = 0.039; Fig. 3). Although birds were more likely to land near the camera during the *training* stage, than the *habituation* stage (Est = 0.76 ± 0.22, z = 3.29, p < 0.001), the experimental stage did not impact social cue usage. Similarly to the food choice model, the observer’s location predicted their food choice, such that birds which chose the risky side of the table were less likely to eat the novel cheese (Est = −0.96 ± 0.30, z = −3.16, p = 0.002). Additionally, sex, age, and trial number, did not impact the observer’s likelihood of landing on the risky side of the table, nor did it impact their use of social information (see Supplemental Table S2).

### Seasonal differences in motivation and risk perception

In both seasons, a greater proportion of individuals avoided consuming the novel test food than avoided foraging near the camera (non-breeding, 88% vs 22%; χ^2^ = 14.57, df = 1, p < 0.001; breeding, 77% vs 27%; χ^2^ = 11.05, df = 1, p < 0.001). Overall, the subset of individuals that participated in both seasons did not become significantly more or less fearful across the year for either type of stimuli (novel food, McNemar’s χ^2^ = 3.13, df = 1, p = 0.077; camera, McNemar’s χ^2^ = 0, df = 1, p = 1).

## Discussion

We found that the type of risk and time of year were critical in determining jackdaws’ use of social cues. Jackdaws were more likely to consume novel foods after witnessing a demonstrator do so throughout the year, but only copied risk-taking demonstrators in approaching a startling object during the breeding season. The greater stability of social cue usage around novel food could arise if birds perceived sampling novel food as risker than approaching a startling object. Consistent with this suggestion, the total number of birds that consumed novel foods was lower than the number that approached the camera, regardless of season. The finding that jackdaws were only influenced by social cues in approaching a startling object during the breeding season suggests that, contrary to our expectations, breeding-related changes in motivation do not result in heightened individual risk-taking in this context. Instead seasonal changes in other factors such as the birds’ social dynamics may generate differences in attention towards conspecifics that could have contributed to the patterns of social cue usage we found.

Jackdaw’s consistently high levels of novel food avoidance provide empirical support for the suggestion that corvids are very neophobic^22^,^31^. The jackdaw population had a comparatively larger percentage of food-wary individuals than what has been reported for populations of other passerine species when the data is compared over a similar number of trials (86–88% vs 26%^32^). Typically, low levels of neophobia are thought to facilitate innovation^7^,^26^, which aids behavioural adjustment to human-induced environmental change^33^. However, corvids counter this trend because they are among the most innovative of birds^23^,^24^, and are often dependent on anthropogenic food sources (e.g. ravens^34^,^35^, jackdaws^36^,^37^), despite being highly neophobic^25^,^31^. Our findings offer a potential route through which corvids may overcome their neophobia. If certain individuals approach novel foods or man-made objects, others can exploit the social information they generate, thereby overcoming their fear.

Despite the potential value of social information, our results demonstrate that the use of social cues to guide behaviour is not consistent, but rather depends critically on the nature of the stimulus and the time of year. Social cues only influenced behaviour around the startling object during breeding season, and it is unclear what aspect of seasonal change generated this pattern. Since more birds were willing to approach the camera than eat the novel foods in both seasons, approaching the camera may have been perceived as a less risky behaviour. With less risk or more motivation to approach risk, one would predict a reduced reliance on social cues. However, we did not find greater overall avoidance of the camera during the breeding versus the non-breeding season, so the seasonal increase in social cue use around objects is unlikely to stem from an increase in wariness of the camera alone. The seasonal effect can also not be attributed to a greater habituation to the camera over time because reduced fear of the camera would in theory have produced greater individual risk taking and lesser use of social cues; an opposite pattern of social cue usage to what we found. Instead, we suggest that the change in social cue usage may be influenced by seasonal changes in attention because jackdaws’ social interactions change across seasons^28^. Although we could not determine the extent to which seasonal changes in group size might have influenced the use of social cues, differences in the number and identity of foraging conspecifics could drive changes in attention towards social instead of non-social cues. Since there are many seasonal behaviours that alter social interactions, such as winter roosting^38^, we suggest such seasonal differences in social cue usage may be common in other species, and could indicate that animals may be better able to adjust to man-made novelty at certain times of year.

In contrast, the wariness and use of social cues around novel food was stable across seasons, but the extent of reliance on social cues depended on the degree of the food’s novelty. We found stronger social cue usage around food in the training versus the test trials. Birds had more experience with the habituation cheese in comparison to the training cheese, than they had with the training cheese in comparison to the test cheese. Therefore the contrast in experience between the two cheese types was much greater during training trials, indicating that the perceived novelty of any stimulus may be a relative consequence of experience, not an absolute one that changes after a single exposure. If the perceived degree of novelty does not fade entirely after a single exposure to a stimulus, then social cues may still be relied upon during subsequent encounters, and could have guided choices during the test phase towards the training cheese.

Since jackdaws responded similarly to social cues around food in both seasons, social influences would likely shape how they exploit anthropogenic foods year round. Although dietary breadth is a predictor of success in urban areas^39^, dietary wariness and its reduction through social learning is not commonly studied in the context of anthropogenic disturbance. However, if individuals overcome their dietary wariness by observing others (e.g.^40^) they may be better equipped to exploit human resources. As jackdaws are reliant on exploiting human-produced food in rural areas^37^ and corvids have been reported to consume human refuse^41^, social learning may be particularly important in allowing them to survive alongside humans.

When social influences increase the likelihood that animals interact with novel and threatening stimuli, social cues may also facilitate learning about such stimuli. Social learning can play a role in spreading human-dependent foraging through populations, thereby increasing human-wildlife conflict (e.g.^42^). However, as we found that not all social cues around novelty are equally influential (i.e. whether observers copied demonstrators’ risk taking depended on the stimulus type, degree of novelty, and time of year), social learning may only occur in certain contexts. Determining where and how social cue usage leads to learning and to novel behaviours is likely to be critical in helping us reduce maladaptive responses to man-made novelty, and mitigate the effects of environmental change.

## Methods

### Study site

Experiments were run in Cornwall, in the South-West of the UK, on free-flying wild jackdaws that were colour ringed following capture in ladder traps or nest boxes as part of the Cornish Jackdaw Project. Two sites, each with two feeding tables were established in locations where humans visit and disturb the area several times an hour: one site in a busy village churchyard (50°11′26″N, 5°10′51″W), the other in an active farmyard (50°11′56″N, 5°10′9″W). The sites were located within 1.5 km of each other. Jackdaws were the main visitor to tables at each site, and the behaviour of other bird species was not analysed. Only one individual was seen at both sites throughout the experiments and was excluded from the study. Sex was determined from a blood sample taken during ringing, and age determined by plumage characteristics or known hatch dates. The population is monitored throughout the year, so the stage of breeding attempts was known. Mild cheddar cheese was used as a reward in experiments at these sites and was familiar to all birds.

### Ethical Statement

Experiments and bird ringing were carried out under approval of Home Office license (PIL 70/25311, PPL to AT 80/2371) and British Trust for Ornithology license (no. C6079, C5752, C5746), and conducted in accordance with the ASAB Guidelines for the Treatment of Animals in Behavioural Research and Teaching^43^.

### Experimental set-up

We conducted the same experiment twice within one year: during the non-breeding season in November to December 2014 (n = 91 trials), and breeding season from April to early May of 2015 (n = 93 trials) while the birds were building nests and laying eggs (see Supplemental Tables 3–4 for trial totals by table and experimental stage). All breeding season trials finished before the first chicks hatched so that birds were choosing food to feed themselves or their partners rather than their chicks.

Each trial consisted of a 90 minute presentation of food alongside a motion-activated camera with flashing lights. Twenty pieces (1 cm^3^) of cheese were placed on a feeding table, split evenly into the 4 corners of the table. The camera was placed approximately 10 cm away from one side of the table, such that two of the food choices were considerably closer to the camera. The camera housing had small red, blue and green lights that flashed repeatedly within 1–2 seconds of a bird’s landing (camera housing dimensions 90 × 80 × 120 mm; Concept Shed, Falmouth, UK). A separate camcorder (Panasonic HC-V130) was set up 20 m away to verify that the motion camera detected all visitors (see videos in Supplementary Information for camera perspective, and examples of behaviour at the table). Each time a bird landed on the table it counted as a visit. Birds visible in the surrounding area were not counted as observers because any birds sitting outside of the camera’s field of view would be missed. However in the few cases where a ringed bird was identified hovering or sitting near the table, it eventually landed on the table.

Cheese was replenished once during the trial if all pieces had been eaten. In trials where cheese of different colours was presented (see stages below), their location was switched at the rebait. Any food remaining on the table at the end of the trial was removed. One trial was run at each of the four tables every day.

### Experimental stages

**Figure 1:**
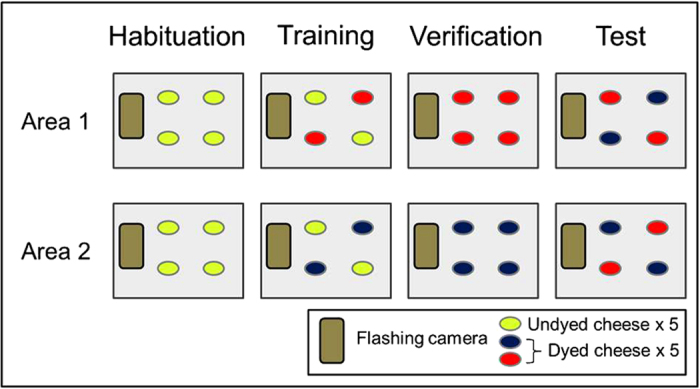
Experimental setup and stages. The cheese was separated into 4 piles of 5 pieces. The habituation stage contained only known, yellow cheese. Training trials contained yellow cheese and a dyed training cheese to allow for a separation of the fear of approaching the food (neophobia) from the fear of consuming it (dietary conservatism). Verification trials ensured birds would forage without the presence of previously known cheese. Test trials determined that responses to each colour were not based on innate avoidance of a particular colour. Each table progressed to the next stage if cheese reliably disappeared from the table for at least three trials in a row. Area 1 and Area 2 were separated by 1.5 km.

**Figure 2:**
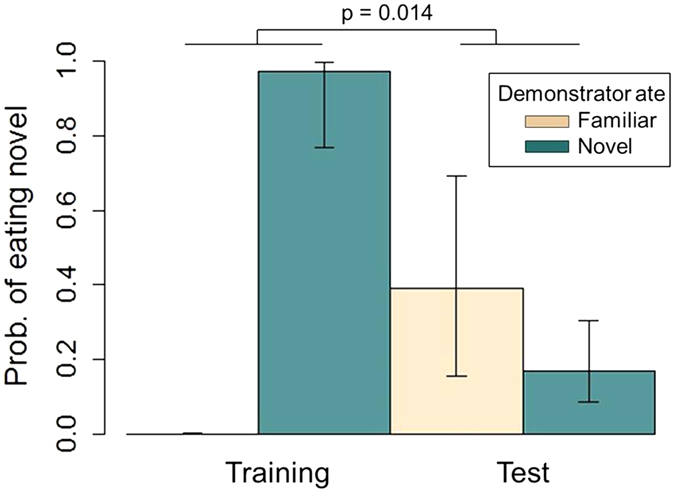
Predicted likelihood of the observer consuming the novel food depending on the demonstrator’s food choice and the stage of experiment. GLMM, n = 212 visits by 44 individuals, Demonstrator_food*Stage, Est = 3.13 ± 1.27 z = 2.46, p = 0.014. Whiskers denote one standard error.

**Figure 3:**
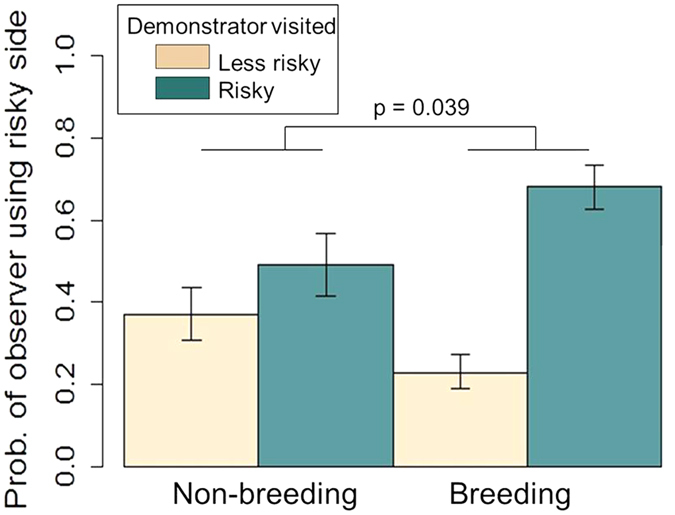
Predicted likelihood of observers using the risky side of the table based on where demonstrators landed and the season. GLMM, n = 516 visits by 85 birds, Demonstrator*Season, Est = 0.86 ± 0.42, z = 2.06, p = 0.039. Whiskers denote one standard error.

The experiment had several stages (adapted from^32^), all which had the motion camera present: *habituation*, *training*, *verification*, and *testing*. In the *habituation* phase we presented plain, undyed yellow cheese, to determine which individuals would eat a familiar food at the table. *Habituation* trials were conducted until no previously unrecorded visitors appeared at the table. *Training* trials offered a novel colour alongside undyed cheese, allowing for the separation of neophobia (fear of approaching the food) from dietary conservatism (reluctance to incorporate novel foods into the diet, *sensu*^44^), as birds feeding on familiar cheese next to the training colour were not deterred by the sight of the novel colour. In the non-breeding season, both tables at one site received red as their training colour, while the other site received blue. In breeding season one site received green and the other black. All four colours have previously been shown to elicit avoidance in captive birds (e.g.^45^). Moreover, the hesitancy jackdaws demonstrated towards them during initial training trials confirmed they were also aversive to wild birds. The experiment moved on to the *verification* stage if all cheese reliably disappeared from the table for at least three trials in a row. *Verification* trials contained only the training colour to ensure that individuals were attracted to this colour without regular cheese present. Finally, in *test* trials, birds were given a choice test containing two piles of their training cheese, and two of a novel cheese colour. The population that received blue for training received red as their novel colour, and vice versa. The same reversal of training and novel cheeses occurred for the breeding season colours. Thus trained colour preferences were group-specific, yet arbitrary (see Fig. 1).

Cheese was coloured by melting and adding food-safe dyes (Sainsbury’s brand). The same number of drops of dye was used for each colour over the course of the experiment. Measurements of each cheese were taken using a spectrometer to verify that birds could discriminate between them. Spectral readings were separated by at least 3.6 just noticeable differences (JNDs; values less than one JND are indistinguishable^46^) and were plotted in the avian visual space using the pavo package in R^47^ (see Supplementary Information).

### Data Analysis

Both motion and camcorder videos were analysed for each trial. For each visit of each trial the following information was recorded: the configuration of cheese on the table when the bird arrived, the visiting bird’s identity, and the colour, amount and location of cheese eaten (either camera side of table or not; see Video 1, 2 for examples of bird behaviour during trials). Food was never knocked off the table by foraging birds, nor did we observe birds stealing food gathered by others, so only individuals visiting the table had the opportunity to feed during the experiment (i.e. there was no scrounging or theft). All bird identities were verified by an additional coder, blind to the stage of the experiment. In the few (n = 16) instances where there was a discrepancy between colour-ring combinations recorded by coders, original videos were consulted and a decision on bird identity was made.

### Statistical Analysis

All data were analysed in R^48^. Birds were deemed to have access to a demonstrator if a conspecific landed to forage at the table less than 30 seconds before their arrival, since foraging groups that we observed tended to gather for longer than this time around the table. As the mere presence of conspecifics has been shown to influence corvids’ levels of object exploration^49^,^50^, observations without demonstrators were removed (401 observations in food analysis, 770 observations in location analysis). This criterion allowed for a comparison of food and location choices based on social information use, not social facilitation. Unringed birds could act as demonstrators, but only individually recognisable, ringed birds were included as observers. Observers’ food choice (novel/familiar) and table side choice (near to/far from the camera) were analysed as separate GLMMs (R package lme4)^51^ with a binomial error structure and logit link function. The food choice analysis only included *training* and *testing* observations when birds had a choice between familiar and novel cheese (169 observations had no food choice). Any birds that had not eaten the familiar cheese prior to that stage were removed (88 observations). The side choice analysis included all experimental stages, but only visits where cheese was available on both sides (262 observations removed). Models investigated the main effects of the observer’s sex and age, the season of the trial, the demonstrator’s choice, the observer’s choice in the other response variable (i.e. their food during side analysis; their side during food analysis) and two-way interactions between all main effects on whether observers chose the riskier option (Y = 1, N = 0). Models also included the potential effects of trial number and experimental stage. Since one table took two more trials than any other to progress past the *training* stage, trial number was capped at the highest number that all tables shared. Bird identity and trial were fitted as random effects. Final models were determined following backwards stepwise elimination of variables based on model AIC values^52^. Effects were retained if their exclusion increased AIC values by at least 2. Once final models had been established, p values and effect sizes of contributing variables were calculated for reporting in text, but all tables refer to changes in AIC (see Supplementary Information).

Differences in social information use between stimuli type or season could arise if approaching the novel food and the camera was differentially risky. To test differences in risk aversion between stimulus types, we conducted chi squared tests on the proportion of birds that never consumed a piece of novel food in test trials, versus the proportion that never foraged on the risky side of the table (near the camera). We also determined whether these were stable traits over the seasons by conducting a McNemar’s chi squared test on the subset of birds (n = 39) that participated at both time points.

## Additional Information

**How to cite this article**: Greggor, A. L. *et al.* Contagious risk taking: social information and context influence wild jackdaws' responses to novelty and risk. *Sci. Rep.* **6**, 27764; doi: 10.1038/srep27764 (2016).

## Supplementary Material

Supplementary Information

Supplementary Video 1

Supplementary Video 2
