# Supplementary material for: Contagious risk taking: social information and context influence wild jackdaws’ responses to novelty and risk
Source: Sci Rep. 2016 Jun 10;6:27764. doi: 10.1038/srep27764 (PMC4901300; doi:10.1038/srep27764)
Supplement: Supplementary Information [file srep27764-s1.pdf]

Contagious risk taking: social information and context influence wild jackdaws' responses to novelty and risk

Alison L. Greggor, Guillam E. McIvor, Nicola S. Clayton, Alex Thornton

Supplementary Information

Colour analysis

To determine whether the cheese colours were visually distinct to the birds, we measured their spectral qualities using an Ocean Optics USB2000 spectrometer, with illumination provided by a PX-2 pulsed Xenon lamp. The probe tip was housed in a hollow sheath so that samples were measured at 45 degrees to normal, and we used a Spectralon 99% white reflectance standard (Labsphere) and a dark current reading to standardize scans. A cheese sample of each colour was measured three times, each at a different location. Colour distances between cheese types were calculated using the coldist function of the pavo package in R [S1, S2], using starlings (*Sturnus vulgaris*) as the visual model, and are reported in units of just noticeable differences (JNDs).

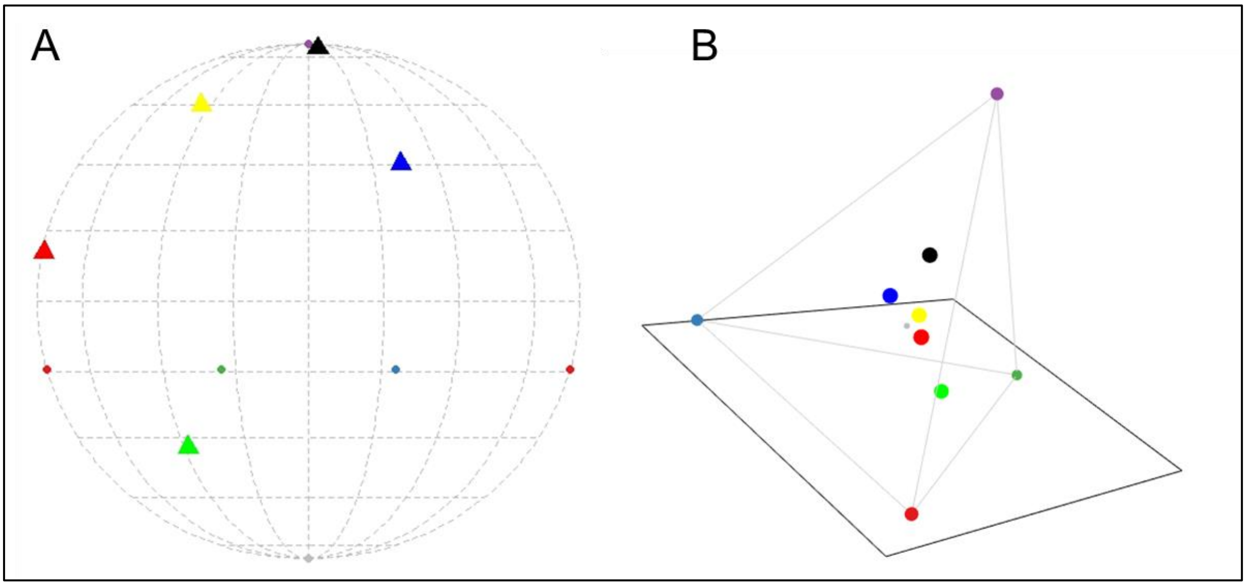

Supplemental Figure S1. Two dimensional (A) and three dimensional (B) projection plot of cheese types in the avian tetrahedral visual space. Both plots show that cheese colours are visually distinct. Cheese colours (red, yellow, green, blue, black) are represented by triangles in (A), by central points in (B). Circles in (A) and triangle vertices in (B) provide reference points to the limit of visible wavelength for each receptor type.

Supplemental Tables S1-S2. Detailed binomial GLMM statistical analysis. Final models are marked in bold, and were reached when no remaining factors could be dropped without AIC values increasing by at least 2. Random effects of bird identity and trial were included in all models. Obs = Observer, Dem = Demonstrator

n = 212 visits, 44 individuals	ΔAIC
Obs_food~ Dem_food + Trial_num+ Stage+ Obs_side + Dem_food*Stage + Age + Dem_side + Season + Dem_food*Season + Dem_food*Trial_num + Sex + Dem_food*Age	0.0
Obs_food~ Dem_food + Trial_num+ Stage+ Obs_side + Dem_food*Stage + Age + Dem_side + Season + Dem_food*Season + Dem_food*Trial_num + Sex	-2.0
Obs_food~ Dem_food + Trial_num+ Stage+ Obs_side + Dem_food*Stage + Age + Dem_side + Season + Dem_food*Season + Dem_food*Trial_num	-1.9
Obs_food~ Dem_food + Trial_num+ Stage+ Obs_side + Dem_food*Stage + Age + Dem_side + Season + Dem_food*Season	-1.7
Obs_food~ Dem_food + Trial_num+ Stage+ Obs_side + Dem_food*Stage + Age + Dem_side + Season	+0.5
Obs_food~ Dem_food + Trial_num+ Stage+ Obs_side + Dem_food*Stage + Age + Dem_side	-1.6
Obs_food~ Dem_food + Trial_num+ Stage+ Obs_side + Dem_food*Stage + Age	0.0
**Obs_food~ Dem_food + Trial_num+ Stage+ Obs_side + Dem_food*Stage**	+1.8

Table S1. Dem_food = Influence of demonstrator's food choice on observer food choice (Novel = 1, Familiar = 0). Dem_side = Influence of demonstrator's table side choice on observer food choice (Scary side = 1, Less scary side = 0). Since no females observed a demonstrator eating a novel cheese, Demonstrator_food:Sex interaction was not included. Age could only just be dropped. When included, younger birds were borderline more likely to eat novel food colours (Est = 1.83±0.93 z = 2.0, p = 0.05).

n = 506 visits, 81 individuals	ΔAIC
Obs_Side ~ Dem_side + Season + Dem_side*Season + Obs_Food + Stage + Dem_food + Age + Dem_side*Age + Trial_num + Dem_side*Trial_num + Sex+ Dem_side*Sex + Dem_side*Stage	0.0
Obs_Side ~ Dem_side + Season + Dem_side*Season + Obs_Food + Stage + Dem_food + Age + Dem_side*Age + Trial_num + Dem_side*Trial_num + Sex+ Dem_side*Sex	-1.7
Obs_Side ~ Dem_side + Season + Dem_side*Season + Obs_Food + Stage + Dem_food + Age + Dem_side*Age + Trial_num + Dem_side*Trial_num + Sex+ Dem_side*Sex	-1.5
Obs_Side ~ Dem_side + Season + Dem_side*Season + Obs_Food + Stage + Dem_food + Age + Dem_side*Age + Trial_num + Dem_side*Trial_num	-1.1
Obs_Side ~ Dem_side + Season + Dem_side*Season + Obs_Food + Stage + Dem_food + Age + Dem_side*Age + Trial_num	+0.1
Obs_Side ~ Dem_side + Season + Dem_side*Season + Obs_Food + Stage + Dem_food + Age + Dem_side*Age	-0.9
Obs_Side ~ Dem_side + Season + Dem_side*Season + Obs_Food + Stage + Dem_food + Age	-0.1
Obs_Side ~ Dem_side + Season + Dem_side*Season + Obs_Food + Stage + Dem_food	-2.0
**Obs_Side ~ Dem_side + Season + Dem_side*Season + Obs_Food + Stage**	+1.0

Table S2. Dem_side = Influence of demonstrator's table side on observer table side. (Scary side = 1, Less scary side = 0). Dem_food = Influence of demonstrator's food choice on observer table side (Novel = 1, Familiar = 0)

Supplementary Table S3. Number of trials run per experimental stage per table in the non-breeding season.

	Habituation	Training	Verification	Test
Church Wall	4	10	1	7
Church Field	4	7	4	6
Farm Coop	5	9	2	9
Farm Silo	4	9	1	9

Supplementary Table S4. Number of trials run per experimental stage per table in the breeding season.

	Habituation	Training	Verification	Test
Church Wall	5	5	3	7
Church Field	6	6	3	7
Farm Coop	5	10	2	7
Farm Silo	6	12	2	7

Video Legends

Video 1. Wild jackdaw displaying fear of man-made camera despite familiar food nearby. Camcorder perspective.

Video 2. Wild jackdaws foraging close and far away from man-made camera during training trial. Motion camera perspective.

Supplementary References

S1. Maia, R., Eliason, C. M., Bitton, P.-P., Doucet, S. M., and Shawkey, M. D. (2013). pavo : an R package for the analysis, visualization and organization of spectral data. Methods Ecol. Evol., n/a–n/a. Available at: http://doi.wiley.com/10.1111/2041-210X.12069 [Accessed September 14, 2015].

S2. Team, R. D. C. (2015). R: A language and environment for statistical computing. Available at: http://www.r-project.org/.
